# Visual appearance of the uterine cervix differs on the basis of HPV type status in high-grade squamous intraepithelial lesion: the results of a reliable method

**DOI:** 10.1186/s12905-021-01565-1

**Published:** 2022-01-30

**Authors:** Qi Zhou, Yingxin Gong, Xiangmei Qiu, Long Sui, Hongwei Zhang, Yan Wang, Lin Lin, Wenjing Diao, Yanyun Li

**Affiliations:** 1 Obstetrics and Gynecology Hospital of Fudan University, 419 Fangxie Road, Shanghai, 200011 China; 2 The First Affiliated Hospital of Zunyi Medical College, Guizhou, 563000 China

**Keywords:** Colposcopy, Cervical cancer, High-grade squamous intraepithelial lesion (HSIL), Human papillomavirus (HPV), ImageJ

## Abstract

**Background:**

This study aimed to evaluate the differences in cervical appearance among different human papillomavirus (HPV) genotypes in patients with high-grade squamous intraepithelial lesions (HSILs).

**Methods:**

A total of 239 histopathological HSIL patients were included and divided into eight groups on the basis of HPV genotype in this prospective study. We present a reliable imaging method that provides reproducible, sensitive and unbiased assessments of cervical appearance characteristics. Colorimetric and morphometric data of colposcopic patterns after the application of acetic acid and iodine were acquired using ImageJ software and the surrounding normal regions were used as controls.

**Results:**

The differences in red, green, blue and mean greyscale values in acetowhite epithelium obtained from ImageJ were not significant between the HPV16 and HPV18 groups (*P *< 0.05). The differences in red, green, and mean greyscale values in iodine staining were significant between the HPV18 and the other groups (*P *< 0.05). The frequency of the occurrence of the coarse mosaic patterns was significantly different among groups (*P *< 0.05), reducing in sequence were the HPV16, HPV-negative, HPV18, HPV31/33 and HPV52/58 groups. For the lesion area of HSILs, the HPV-negative group was the largest. The sensitivity of colposcopic impression varied among HPV genotypes (*P *< 0.01), being lowest in the HPV52 group.

**Conclusions:**

Although being nonspecific, iodine negativity should be concerned in HPV18-positive lesions which is closely related to glandular epithelium. Vascular patterns in HPV52/58-positive HSIL are quite occult and tend to be missed by colposcopists. HPV-negative lesions are prone to be large and present typical vascular patterns despite being rare.

## Background

Globally, cervical cancer is the most common gynaecological malignancy, and in many countries, the incidence of cervical cancer is still increasing in recent years [1–4]. Most cervical cancers and cervical intraepithelial neoplasias are attributable to persistent infection with high-risk human papillomavirus (HPV), among which types HPV16 and HPV18 account for 70% of all cases and other high-risk HPV types (HPVs 31/33/52/58, etc.) account for 30% [4–7]. Currently, colposcopy-guided biopsy with histopathology is considered the gold-standard method for the diagnosis of cervical cancer and cervical intraepithelial neoplasia [8, 9]. By applying acetic acid (3–5%) and Lugol’s iodine, a colposcopist can detect abnormalities in cervical visual appearance based on changes in the epithelium and blood vessels [10]. As colposcopy is a subjective procedure, some colposcopic scoring and nomenclature systems have been introduced worldwide [11–13]. Among them, the latest and most popular is the International Federation of Cervical Pathology and Colposcopy (IFCPC) nomenclature published in 2011, which was also the important basis for the 2018 American Society for Colposcopy and Cervical Pathology (ASCCP) Colposcopy Standards recommendations. In the modified IFCPC nomenclature, colposcopic findings are classified and described in detail, and most of them are based on typical visual appearance, especially those related to HPV16 and HPV18 [11, 14–16].

For all the systems, colposcopic findings are classified according to the severity of cervical lesions, such as normal/benign lesions, low-grade squamous intraepithelial lesions (LSILs), high-grade squamous intraepithelial lesions (HSILs), and invasive lesions. However, the correlation between colposcopic findings and HPV types has never been examined [13, 14]. Some research studies have evaluated the different appearances of the cervix caused by different HPV genotypes, but the results were inconsistent [17]. Some studies showed that a definite lesion was present when HPV DNA was found, particularly HPV16, regardless of histological diagnosis. HPV16 was associated with more prominent colposcopic abnormalities and larger lesions than other oncogenic HPV types, and this appearance could be used to improve risk stratification and facilitate lesion detection by colposcopy [18–20], while HPV18/45-related precancerous lesions were found to be more difficult to detect by existing cytological and colposcopic techniques than HPV16-related lesions [21]. In contrast, Jeronimo’s study showed that the link between HPV infection status and visual appearance of the cervix was weak for each individual colposcopist [22]. Colposcopic findings were considered to be mainly related to the severity of cervical dysplasia [23]. However, all the conclusions in the above studies were made based on the subjective evaluation of observers. To date, there has been no report on the quantitative analysis of colposcopic images to evaluate the correlation of the visual appearance of the cervix and different HPV genotypes.

In recent years, HPV vaccines have been introduced in many countries worldwide [24, 25]. It is believed that HPV vaccination should result in a reduction in the proportion of cervical abnormalities resulting from infection by HPV types 16 or 18, reducing the prominence of lesions [26–28]. ImageJ, a free image analysis software developed by the National Institute of Health (NIH), possesses high agreement by analyzing the pixel composition of digital image, and has been widely used in many scientific research fields. In this study, we utilized ImageJ analysis software to objectively quantify colposcopic findings to evaluate the differences in cervical appearance caused by the different HPV types in patients with cervical HSILs.

## Methods

### Study design and population

In this observational and prospective cohort study, from January 2020 to December 2020, the population of patients with clinically suspected cervical intraepithelial neoplasia (CIN) who underwent colposcopy-guided punch biopsy of the cervix and the detection of HPV genotypes at the Obstetrics and Gynaecology Hospital of Fudan University were enrolled, with a total of 2816 patients. The inclusion criteria for clinically suspected CIN were as follows: abnormal cytological results, positive high-risk HPV testing, or suspicious clinical manifestations. Abnormal cytological results included atypical squamous cells of undetermined significance (ASC-US) or worse. The exclusion criteria were as follows: (1) patients with undefined HPV genotype results; (2) patients with low-risk HPV genotype results; (3) some unfavourable factors for colposcopic diagnosis, such as cervical histopathological results obtained within 1 year, previous cervical surgery, pelvic radiotherapy, hysterectomy, pregnancy, and inadequate colposcopic examination; and (4) patients with incomplete data, such as unqualified colposcopic images, undefined histopathological diagnosis, and incomplete history records. Finally, a total of 239 patients with cervical HSILs diagnosed by histopathology were included in this study. All patients signed informed consent forms before the study, and institutional review board approval was obtained (2020-24).

In order to balance the sample size in each group, the grouping was based on the incidence rate of HPV oncogenic types in cervical lesions in China latest published by ICO / IARC (www.hpvcentre.net, 22/01/2019). According to the HPV types, all the patients were grouped as follows: the HPV16 group (single type 16 or multiple types including 16 but excluding 18), the HPV18 group (single type 18 or multiple types including 18 but excluding 16), HPV31/33 group (single type 31 or 33), HPV52 group (single type 52), HPV58 group (single type 58), other single-type HPV group (including carcinogenic types 35, 39, 45, 51, 53, 56, 59, 66, 68, 26, 73, or 82), other multiple-HPV group (more than one carcinogenic HPV type, excluding 16 and 18) and HPV negative group. Differences in the visual appearance of the cervix in each group were compared by evaluating the colposcopic images. The sample size of each group was at least 16.5 patients, which was calculated based on the frequency of thin acetowhite epithelium in the HPV16 group and HPV-negative group being 60% and 30%, respectively, in the preliminary study, with 5% Type I error and 5% Type II error accepted.

### Detection of HPV

Cervical samples for HPV testing were collected by using a cytobrush. The Roche Cobas HPV Test (Roche Molecular Systems, Pleasanton, California) and the HPV Genotyping Real Time PCR Kit (Jiangsu Bioperfectus Technologies, Jiangsu, China) were used to detect high-risk HPV genotyping. The Roche Cobas HPV Test detects (without quantification) HPV16, HPV18, and other high-risk HPV types (types 31, 33, 35, 39, 45, 51, 52, 56, 58, 59, 66, and 68). The HPV Genotyping Test can detect each HPV type among 18 high-risk types (types 16, 18, 31, 33, 35, 39, 45, 51, 52, 53, 56, 58, 59, 66, 68, 26, 73, 82) and 3 low-risk types (types 6, 11, 81).

### Colposcopy

All colposcopies were performed and recorded by experienced colposcopists who had more than 5 years of experience in colposcopic diagnosis with the 2011 IFCPC nomenclature. A Leisegang BG/LED Y/C optoelectronic integrated digital colposcopy system (Leisegang Feinmechanik Optik GmbH, Berlin, Germany) was used to observe colposcopic patterns, and images were obtained by an optical camera (Canon EOS600D). A standard colposcopy protocol, including the application of 3% acetic acid and Lugol’s iodine solution for the Schiller test, was followed to ensure that uniform, high-pixel, and multistage colposcopic images could be obtained. The procedure of collecting images was as follows: (a) Initial state: the cervix was exposed and an image was taken at 7.5 times magnification in Position I, where the cervix could be fully exposed in the image with the os in the centre; (b) After saline: an image was taken at 7.5 times magnification in Position I; (c) 30 s after 3% acetic acid: an image was taken at 7.5 times magnification in Position I; (d) 60–90 s after 3% acetic acid: an image was taken at 15 times magnification in Position II, where the anterior lip of the cervix was completely exposed and the os was almost at the bottom of the image, and an image was taken at 15 times magnification in Position III, where the posterior lip of cervix was completely exposed and the os was almost at the top of the image, and then an image was taken at 15 times magnification in Position IV, where the os was in the image centre; (e) 120–180 s after 3% acetic acid: an image was taken at 15 times magnification in Position II, Position III and Position IV; (f) 240 and 300 s after 3% acetic acid: images were taken at 7.5 times magnification in Position I; and (g) After Lugol’s iodine: an image was taken at 7.5 times magnification in Position I. A total of 12 images were collected for each patient, with a format of JPGE, a resolution of 6000 × 4000 pixels, a horizontal resolution of 96 dpi, a vertical resolution of 96 dpi, and a bit depth of 24.

### Image evaluation

A total of 239 patients with 2868 colposcopic images were assigned randomly to six experienced colposcopists for evaluation. Each colposcopist evaluated a set of 956 images to ensure that each image was evaluated by at least two colposcopists. Evaluations were performed according to the 2011 IFCPC nomenclature, which was documented as follows [14]. (1) General assessment: adequate or inadequate for the reason, squamocolumnar junction visibility, and transformation zone types. (2) Normal colposcopic findings: original squamous epithelium, columnar epithelium, metaplastic squamous epithelium, deciduosis, and location and size of lesions. (3) Abnormal colposcopic findings: thin acetowhite epithelium (AWE), fine mosaic and fine punctuation as minor changes; dense AWE, coarse mosaic, coarse punctuation, sharp border, inner border sign, and ridge sign as major changes; leukoplakia, erosion, and iodine negativity as nonspecific changes; atypical vessels and additional signs (exophytic lesion, necrosis, ulceration, etc.) suspicious for invasion changes; condyloma, polyps, and obvious contact bleeding as miscellaneous findings. (4) Finally, the colposcopists recorded the description of colposcopic findings and made a final diagnosis, classifying the findings as a normal/benign lesion, LSIL, HSIL, or carcinoma. If there was disagreement between colposcopists, a consensus was reached through discussion.

The colposcopist was blinded to the biopsy results and HPV infection status during the colposcopic procedures. All the evaluators were blinded to any clinical data, including HPV status and colposcopic diagnosis.

### ImageJ software analysis

**Fig. 1:**
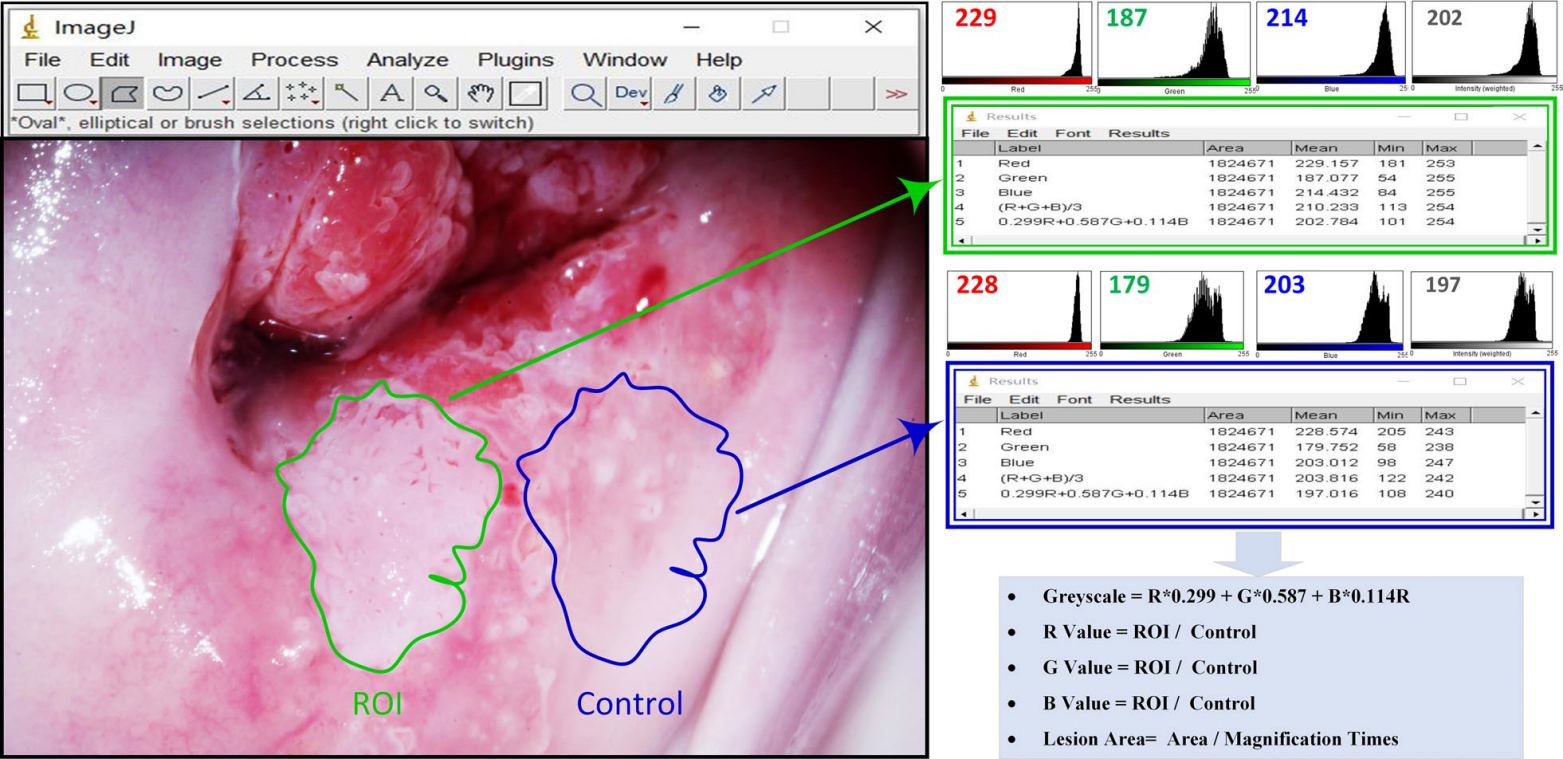
Digital image analysis of acetowhite epithelium. ROI (green outline) and control (blue outline) are selected and the values of red, green, and blue are measured by using ImageJ software. The mean greyscale value is calculated according to the formula (Grey = R*0.299 + G*0.587 + B*0.114). The number in the top left corner of each colour histogram corresponds to the mean colour value of red, green, blue and greyscale

To quantify the colposcopic findings objectively, we used ImageJ software, a free image analysis software developed by the NIH. The image with the most obvious acetowhite epithelium and the image with the largest lesion (and most complete) were selected from among the 12 images of each patient. These images were saved in JPEG format and subsequently transferred to ImageJ photo analysis software. After training the ImageJ Standard Operating Procedure (SOP), the colposcopist selected the region of interest (ROI) for each image. Then, colposcopic findings were qualified and analysed using various statistical moments for a finer analysis, as shown in Fig. 1.

1. Quantitative analysis of acetowhite epithelium

The reaction of acetic acid solution with nuclear protein and keratin in cells results in reversible protein coagulation, which is white when observed under colposcopy. Because of different indices of refraction, which are associated with the density of epithelial cells, the light intensity of acetowhite epithelium in the image is different. The RGB colour model, as a kind of colour representation, is the most commonly used model for digital colour images. For an RGB colour image, the intensity of light in the red, green, and blue channels can be captured by three colour sensors per pixel and then analysed [29]. The mean greyscale value, which is calculated according to the formula (Grey = R*0.299 + G*0.587 + B*0.114), is used to provide a measure of average intensity. Based on the above theory, the two colposcopists used freehand selection to draw a boundary around the most striking feature of acetowhite epithelium, which was defined as an ROI. ImageJ software was used to calculate the mean R, G, and B and the mean greyscale values at the ROIs at the same time. Close to the lesion, a similar-sized normal epithelial region was selected as a reference in the same way. The final R value obtained, which was the average of the results measured by the two colposcopists, was the ratio of the lesion area’s R value to the normal area’s R value, and the same was true with G, B, and mean greyscale values.

(2) Quantitative analysis of iodine staining

Based on the acetowhite epithelium ROI selected before, the two colposcopists also used freehand selection to draw a boundary around the most striking feature of iodine negativity. Similarly, a normal epithelial region with iodine staining was selected as a reference. The final R, G, B, and mean greyscale values were obtained by using the same method as the acetowhite epithelium.

(3) Quantitative analysis of lesion area

Based on the acetowhite epithelium, the two colposcopists used freehand selection to draw a boundary around the most suspicious lesion area, and the area value was measured by ImageJ. The final value was obtained from the ratio of the lesion area measured by ImageJ divided by the magnification of the image.

### Histopathology

Colposcopy-guided punch biopsies were performed at the suspicious areas. Histopathological diagnoses, classified as normal or benign lesions, LSILs, HSILs, or carcinomas according to the 2012 Lower Anogenital Squamous Terminology (LAST) [30], were made by two senior gynaecological pathologists in our hospital.

### Statistical analysis

Statistical analysis was performed using the Statistical Package for Social Sciences Version 20.0 Software (SPSS 20.0). Age data are expressed as the mean ± standard deviation (SD). If the variance was homogeneous, one-way analysis of variance was used to compare the differences in quantitative variables. If the variance was not homogeneous, the Welch test was used. The Spearman rank correlation coefficient (*r*_*s*_) was used to assess the correlation among red, green, blue, and greyscale values. The chi-squared test or Kruskal–Wallis test was used to compare the differences between different categorical variables. The accuracy of diagnostic methods was evaluated by the sensitivity and specificity. Any P value less than 0.05 was considered to indicate a statistically significant result.

## Results

### Patient characteristics

A total of 239 patients with cervical HSILs diagnosed by histopathology at the Obstetrics and Gynaecology Hospital of Fudan University from January 2020 to December 2020 were included in this study. All patients were divided into eight groups according to HPV genotypes as mentioned in the Materials and Methods. The number of patients, mean age, and cytological findings in the different groups are detailed in Table 1. The proportions of patients in the HPV16, HPV18, HPV31/33, HPV52, HPV58, and other single/multiple HPV- and HPV-negative groups were 36.4% (87/239), 7.5% (18/239), 9.2% (22/239), 19.2% (46/239), 20.5% (49/239), and 7.1% (17/239), respectively. The mean age of all patients was 42.4±11.8 years (range: 21–78 years). The mean age of patients in the HPV16 group was 37.5±10.2, which was the lowest among all groups. The mean age of patients in the multiple HPV group was 48.6±12.9 years, which was the highest. The differences in the mean age among the eight groups were significant (*P *< 0.001). The incidence rate of notably abnormal cytological findings (>LSIL) was 65.2% (15/23) in the HPV58 group, which was the highest among the groups, followed by 61.1% (11/18) in the HPV18 group. However, the differences among the eight groups were not significant (*P *= 0.267).

**Table 1:** The mean age, cytological distribution and colposcopic impression in the different HPV genotype groups

	HPV genotype groups								*P*
	HPV 16	HPV 18	HPV 31/33	HPV 52	HPV 58	Other single HPV	Other multiple HPVs	HPV negative	
Age (years)	37.5 ± 10.2	43.8 ± 7.7	43.3 ± 13.5	41.2 ± 10.3	45.8 ± 9.4	46.9 ± 12.6	48.6 ± 12.9	46.4 ± 14.4	< 0.001^e^
*Cytology* (n = 239)									
≤ LSIL^a^	49	7	11	16	8	12	18	10	0.267^f^
> LSIL^b^	38	11	11	7	15	7	12	7	
*Colposcopic impression* (n = 239)									
≤ LSIL^c^	13	4	5	11	7	9	11	8	0.006^ g^
> LSIL^d^	74	14	17	12	16	10	19	9	
Total (n)	87	18	22	23	23	19	30	17	

^a^Including negative for intraepithelial lesion or malignancy (NILM), atypical squamous cells of undetermined significance (ASC-US) and low-grade squamous intraepithelial lesions (LSILs)

^b^Including atypical squamous cells, cannot exclude high-grade squamous intraepithelial lesions (ASC-H); low-grade squamous intraepithelial lesions, cannot exclude high-grade" (LSIL-H); high-grade squamous intraepithelial lesions (HSILs); atypical glandular cells (AGC); and adenocarcinoma in situ (AIS)

^c^Including normal or benign lesions and LSILs

^d^Including HSILs and carcinomas

^e^Welch test for the comparison of age among different groups

^f^Kruskal–Wallis H test for the comparison of cytological distribution among different groups

^g^Kruskal–Wallis H test for the comparison of the sensitivity of colposcopic impression among groups

### Quantitative analysis of acetowhite epithelium and iodine staining by Image J

**Fig. 2:**
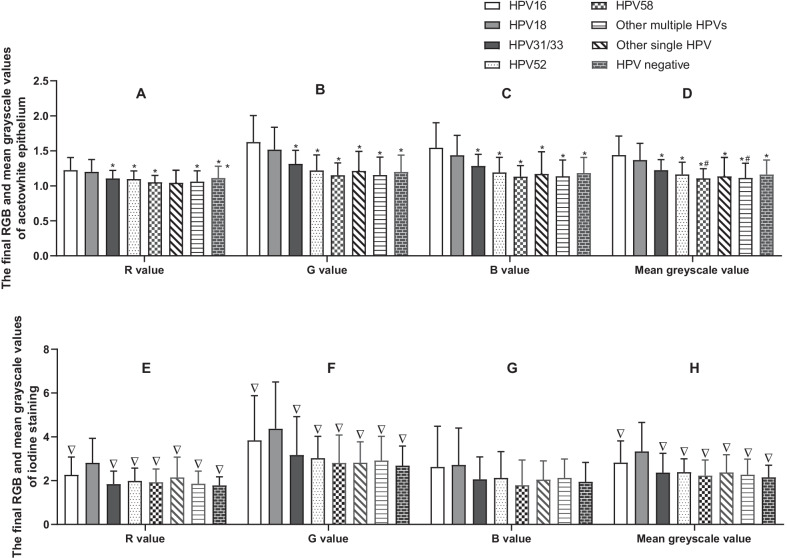
The final RGB and mean greyscale values (the ratio of the lesion area value to the normal area value) of acetowhite epithelium (**A**–**D**) and iodine staining (**E**–**H**) in the eight groups. Data are expressed as the means ± SEM. *P<0.05, compared with the HPV16 group; #*P *< 0.05, compared with the HPV18 group; ▽*P *< 0.05, compared with the HPV18 group

Image J was applied to quantify the acetowhite epithelium, and iodine staining was objectively performed by calculating the red (R), green (G), blue (B), and mean greyscale values based on the ratio of the lesion area value to the normal area value. Figure 2 shows the differences in the final R, G, B and mean greyscale value measurements in acetowhite epithelium (A–D) and iodine staining (E–H). For acetowhite epithelium, the HPV16 group had the highest R, G, B, and mean greyscale values among the groups. The differences between the HPV16 group and the HPV18 group were not significant (*P* > 0.05 in R, G, B and mean greyscale value), while the differences between the HPV16 group and the other six groups were significant (*P* < 0.05 in R value, *P *< 0.001 in G, B and mean greyscale values). The mean greyscale value of the HPV18 group was significantly different from that of the HPV58 group and multiple HPVs group (*P* < 0.05). For iodine staining, the HPV18 group had the highest R, G, and mean greyscale values among the groups. The differences in R, G and mean greyscale values between the HPV18 group and the other groups were significant (*P* < 0.05), while the differences in R, G, B and mean greyscale values among the other groups were not significant (*P* > 0.05).

### Correlations between RGB values and mean greyscale values

To understand the correlation of red, green, and blue values and the mean greyscale value in acetowhite epithelium and iodine staining, Spearman correlation analysis was applied, as shown in Table 2. The R, G, and B values were all positively correlated with the mean greyscale value (*P*<0.001). For acetowhite epithelium, the Spearman correlation coefficients between the R, G, and B values and the mean greyscale value were 0.932, 0.989, and 0.978, respectively. For iodine staining, the Spearman correlation coefficients between the R, G, and B values and the mean greyscale value were 0.830, 0.939, and 0.633, respectively. The correlation between the green value and the mean greyscale value was more significant than that between both the red and the blue value and the mean greyscale value in both acetowhite epithelium and iodine staining.

**Table 2:** Correlation between RGB values and mean greyscale value in acetowhite epithelium and iodine staining

	Correlation (n = 239)	Mean grayscale value
*Acetowhite epithelium*		
Red value	*r*_*s*_^a^	0.932
	*P*^b^	< 0.001
Green value	*r*_*s*_^a^	0.989
	*P*^b^	< 0.001
Blue value	*r*_*s*_^a^	0.978
	*P*^b^	< 0.001
*Iodine staining*		
Red value	*r*_*s*_^a^	0.830
	*P*^b^	< 0.001
Green value	*r*_*s*_^a^	0.939
	*P*^b^	< 0.001
Blue value	*r*_*s*_^a^	0.633
	*P*^b^	< 0.001

^a^The coefficient of Spearman correlation

^b^Correlation is significant at the 0.01 level (2-tailed)

### Colposcopic vascular patterns in different HPV type groups

The frequency of the occurrence of five vascular patterns (fine mosaic, fine punctuation, coarse mosaic, coarse punctuation, and atypical vessels) in the eight groups was calculated, and the differences among groups were analysed by Pearson chi-square test (Table 3). The frequency of the occurrence of the fine punctuation pattern among different groups was significantly different (*P *= 0.032), and it decreased successively in the HPV 31/33 group, HPV 52 group, HPV 58 group, HPV 16 group, other multiple HPV group, HPV 18 group, other single HPV group, and HPV-negative group. The frequency of the occurrence of the coarse mosaic pattern among different groups was also significantly different (*P *= 0.049), and it decreased successively in the HPV 16 group, HPV-negative group, HPV 18 group, HPV 31/33 group, HPV 52 group, other multiple HPV group, HPV 58 group, and other single HPV group. There were no significant differences among the groups in the frequency of the occurrence of the other three vascular patterns (*P *> 0.05).

**Table 3:** Colposcopic vascular patterns in the groups with different HPV genotypes

Vascular patterns	HPV genotype groups (patient)								P^*^
	HPV16 (%)	HPV18 (%)	HPV31/33 (%)	HPV52 (%)	HPV58 (%)	Other single HPV (%)	Other multiple HPVs (%)	HPV negative (%)	
Fine mosaic	6 (6.9)	1 (5.6)	4 (18.2)	6 (26.1)	4 (17.4)	3 (15.8)	5 (16.7)	0	0.111
Fine punctuation	10 (11.5)	0 (0.0)	5 (22.7)	5 (21.7)	3 (13.0)	0 (0.0)	1 (3.3)	0	0.032
Coarse mosaic	35 (40.2)	6 (33.3)	7 (31.8)	6 (26.1)	3 (13.0)	2 (10.5)	5 (16.7)	6 (35.3)	0.049
Coarse punctuation	33 (37.9)	5 (27.8)	7 (31.8)	8 (34.8)	5 (21.7)	4 (21.1)	6 (20.0)	9 (52.9)	0.241
Atypical vessels	6 (6.9)	2 (11.1)	1 (4.5)	0	0	0	0	2 (11.8)	0.229
Total	87	18	22	23	23	19	30	17	

^*^Pearson chi-square test for the comparison of the occurrence of colposcopic vascular patterns among groups

### Quantitative analysis of the lesion areas

**Fig. 3:**
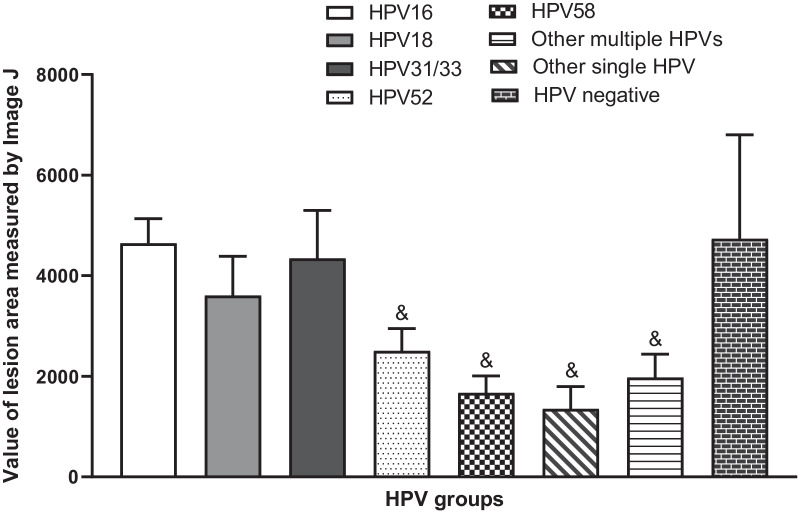
The value of the lesion area in the eight groups. Data are expressed as the means ± SEM. &*P *< 0.05, compared with HPV16 group

We applied ImageJ to analyse the lesion areas in the eight groups, and the final value was obtained from the ratio of the lesion area measured by ImageJ divided by the magnification of the image (Fig. 3). The lesion area of the HPV16 group was significantly different from that of the HPV52 group (*P *= 0.035), HPV58 group (*P *= 0.003), other single HPV group (*P *= 0.002), and other multiple HPV group (*P *= 0.003). The largest lesion area was in the HPV-negative group, the second largest was in the HPV16 group, and the smallest lesion area was in the other single HPV group.

### Colposcopic impression in different HPV type groups

The sensitivity of colposcopic impression in the diagnosis of HSILs in different HPV type groups was analysed (Table 1). The sensitivity was significantly different among the groups (*P *= 0.006), decreasing successively in the HPV16 group (74/87, 85.1%), HPV18 group (14/18, 77.7%), HPV31/33 group (17/22, 77.3%), HPV58 group (16/23, 69.6%), other multiple HPV group (19/30, 63.3%), HPV-negative group (9/17, 52.9%), other single HPV group (10/19, 52.6%) and HPV52 group (12/23, 52.2%).

## Discussion

To our knowledge, this is the first prospective study evaluating the differences in cervical appearance caused by different HPV genotypes in HSIL patients based on an objective and quantitative approach. Through 2868 colposcopic images from 239 histopathological cervical HSIL patients that were divided into eight groups on the basis of HPV genotype, we found that some major findings in colposcopy, such as acetowhite epithelium, iodine staining, and vascular pattern, were typical in the HPV16 or HPV18 group, while they were occult in other groups, especially for some locally prevalent genotypes, such as HPV52 or HPV58. Although some previous reports showed that colposcopic findings were mainly correlated with histopathological results, our findings provided evidence for the correlation between HPV genotypes and colposcopic findings.

Our study found that the mean age of patients in the HPV16 group was 37.5 years, which was significantly younger than that of patients in the other genotype groups (ranging from 41.2 to 48.6 years). This finding was in line with that of Song et al. [31] that the 29–39 age group was at the highest risk of cervical HSILs or worse compared with older age groups of HPV16-positive women, and the 40–49 age group was at the highest risk among other-hrHPV-positive women, which was also supported by Argyri [32] and Aro [33]. These results suggested that the cervical lesions in HPV16-positive women might develop into HSILs at a younger age or be diagnosed earlier than the cervical lesions in women with other hrHPV genotypes. Some studies reported a correlation between cervical HPV genotypes and cytological findings in the general population, including the latest study in our clinical centre and another publication revealing the substantial correlation between eight HPV genotypes and cytological HSILs or worse [34, 35]. However, few publications report this correlation in specific histopathological HSIL populations. Our study found no significant difference among different HPV-type-infected patients under the same histopathological diagnosis of HSILs, implying the potential consistency between cervical histopathology and cytology, regardless of HPV genotype.

Vascular patterns and epithelial signs are the main manifestations in the colposcopic diagnosis of cervical lesions, which was exhibited in the 2011 IFCPC nomenclature. In this study, epithelial signs under acetic acid and iodine staining were quantified by ImageJ, and it was found that the acetowhite epithelium in HPV16-positive HSIL women was typical, with significantly higher R, G, B, and mean greyscale values than that in other-HPV-type positive women. This result supports the findings from two previous studies in which visual inspection with acetic acid was found to be more sensitive in HPV16-positive women than in non-HPV16-infected patients [22, 36], which suggested that HPV16-related HSILs were easier to detect by colposcopy. For iodine staining, HPV18-related HSILs showed a specific appearance, among which R, G, and mean greyscale values were significantly highest among all HPV types. The possible explanation is that HPV18 tends to infect the glandular epithelium, which lacks glycogen and usually exhibits iodine staining negativity [37]. For the colour characteristics of acetowhite epithelium and iodine staining, we confirmed that the R, G, B values were all positively correlated with the mean greyscale value, especially the green value, which was the most significant in both acetowhite and iodine staining. The present findings suggested that typical acetowhite epithelium is mostly related to HPV16-positive lesions, and HPV18-positive lesions usually exhibit iodine staining negativity, while the lesions for other HPV types are all atypical in both acetowhite and iodine staining.

We then further analysed the vascular patterns and showed that the frequencies of the occurrence of the fine punctuation pattern in grade 1 findings and the coarse mosaic pattern in grade 2 findings were significantly different among women with different HPV genotype infections. Grade 2 coarse mosaic patterns were significantly more common in HPV16- and HPV18-positive women than in other-HPV-type-positive women, especially HPV52/58-positive women, in whom vascular patterns were quite occult. Interestingly, HPV-negative HSIL patients showed typical grade 2 vascular patterns, second only to HPV16-positive women, which was unexpected but might imply a characteristic of HPV-negative HSILs. This study is the first aiming at identifying the differences in colposcopic vascular patterns among patients with different HPV genotypes, and it was found that HPV genotype might be one of the main factors that lead to abnormal vascular patterns even under the same histopathological diagnosis.

Nam’s and Chen’s studies showed that larger colposcopic lesions (≥ 3 quadrants involved) were significantly associated with HPV16 [18, 38], while another report showed that lesion size was not related to HPV16 status [19]. Likewise, our study found that HPV16-related HSILs were large, followed by HPV31/33- and HPV18-related lesions, which were significantly larger than the lesions of other HPV types, including HPV52/58. Interestingly, similar to the grade 2 vascular pattern, HPV-negative HSILs were found to be particularly large in this study, which implied that HPV-negative lesions are prone to present typical vascular patterns and large lesions despite being rare. A similar situation was found in one study on vulvar squamous cell carcinoma in that HPV-negative tumours were found to be larger in length and width than HPV-positive tumours [39], which suggested two different pathways of oncogenesis due to different HPV statuses.

The colposcopic impression diagnosis made on the basis of subjective judgement is reflective of cervical appearance. However, few publications have reported discrepancies between different HPV genotypes, and the correlation between colposcopic findings and HPV genotypes is controversial [13, 14]. In this study, we found that the sensitivity of colposcopic impression in the detection of cervical HSILs was significantly different among different HPV type groups; it was the highest in HPV16-positive patients, followed by HPV18-positive patients, and was the lowest in HPV52-positive patients. Our findings were consistent with those of Jeronimo et al. that HPV16/18-infected women had worse colposcopic impressions than non-HPV16/18-infected patients [36], resulting in the early diagnosis of HPV16-related CIN3+ and delayed diagnosis in HPV18/45 and other hrHPV-related CIN3+ patients [21, 22].

The factors that were possible limitations in our study were the sample size and the selection bias, as all patients included were from the same hospital. However, we believe that these evaluations are clinically relevant since we analysed the colposcopic findings quantitatively and objectively and compared the differences in visual appearance in different HPV-genotype-related lesions. Similar to Jeronimo’s opinion [22], we consider that the visual appearance of an intraepithelial lesion associated with HPV16 may be more typical, especially after acetic acid is applied, and more likely to be detected under colposcopy. The difference in iodine staining negativity between the HPV18 and other hrHPV groups may further suggest that HPV18 is closely related to glandular epithelial lesions. Lesions associated with HPV52 or HPV58 could be atypical and easier to miss during colposcopic evaluation. It is important to note that the typical vascular patterns and large areas may be characteristics of lesions associated with HPV negativity.

It is estimated that HPV vaccination results in a significant reduction in the prevalence of cervical infection by HPV type 16 or 18, reducing the prominence of cervical lesions [40]. These data provide insight into the performance of colposcopy in vaccinated populations in the future, especially in HPV-negative patients.

## Data Availability

The data that support the findings of this study are available from Obstetrics and Gynecology Hospital of Fudan University but restrictions apply to the availability of these data, which were used under license for the current study, and so are not publicly available. Data are however available from the correspondence author upon reasonable request and with permission of Obstetrics and Gynecology Hospital of Fudan University.

## Abbreviations

HPV: Human papillomavirus
HSIL: High-grade squamous intraepithelial lesion
IFCPC: International Federation of Cervical Pathology and Colposcopy
ASCCP: American Society for Colposcopy and Cervical Pathology
LSIL: Low-grade squamous intraepithelial lesions
CIN: Cervical intraepithelial neoplasia
ASC-US: Atypical squamous cells of undetermined significance
AWE: Acetowhite epithelium
NIH: National Institute of Health
SOP: Standard Operating Procedure
ROI: Region of interest
LAST: Lower Anogenital Squamous Terminology
